# A novel envelope mediated post entry restriction of murine leukaemia virus in human cells is Ref1/TRIM5α independent

**DOI:** 10.1186/1742-4690-7-81

**Published:** 2010-10-07

**Authors:** Nidia MM Oliveira, Roochi Trikha, Áine McKnight

**Affiliations:** 1 HIV/AIDS Group, Centre for Immunology and Infectious Disease, Blizard Institute of Cell and Molecular Science, Barts and the London School of Medicine and Dentistry, 4 Newark Street, Whitechapel, London E1 2AT, UK

## Abstract

**Background:**

'Intrinsic' resistance to retroviral infection was first recognised with the Friend virus susceptibility gene (Fv1), which determines susceptibility to murine leukaemia virus (MLV) infection in different murine species. Similarly, the tripartite motif (TRIM) family of proteins determine lentiviral restriction in a primate host-species specific manner. For example rhesus TRIM5α (rhTRIM5α) can potently restrict HIV-1 infection while human TRIM5α (huTRIM5α) only has a mild effect on SIVmac and HIV-1 infectivity (Lv1). Human TRIM5α is able to restrict MLV-N virus replication, but is ineffective against MLV-B or MLV-NB virus infection. Lv2 restriction of some HIV-2 viruses is seen in human cells. Like Lv1, Lv2 is a post-entry restriction factor, whose viral determinants have been mapped to the viral capsid (CA). Unlike Lv1, however, Lv2 is determined by envelope (Env) in addition to CA. Here we present evidence of a novel Env determined post entry restriction to infection in human cells of pseudotyped MLV-B and MLV-NB cores.

**Results:**

We generated retroviral vectors pseudotyped with various gamma and lentiviral Envs on MLV-B and -NB CAs containing a green fluorescent protein (GFP) reporter. Flow cytometry was used to determine transduction efficiencies in NP2/CD4/CXCR4 (glioma cell line stably transduced with the HIV receptors) and HeLa/CD4 cell lines. The HeLa/CD4 cell line restricted both MLV CAs in an Env dependent manner, compared to NP2/CD4/CXCR4 cells. Quantitative polymerase chain reaction (QT-PCR) analysis of reverse transcription (RT) transcripts demonstrates that this restriction occurs at a post entry and RT level. siRNA knockdown of huTRIM5α ruled out a direct role for this cellular component in mediating this restriction. We describe a previously unobserved Env determined restriction of MLV-B and MLV-NB CAs in HeLa/CD4 cells when pseudotyped with HIV-2 and RD114 Envs, but not gibbon ape leukaemia virus (GALV), HIV-1 or Amphotrophic (Ampho) Envs.

**Conclusions:**

Our data further demonstrate the variability of Env and CA mediated susceptibility to post entry host cell restriction. We discuss the relevance of these findings in light of the growing evidence supporting the complexities involved in innate host immunity to retroviral infection.

## Background

Retroviruses can cause a variety of diseases in their host species. Over-expression, integration near oncogenic loci, or the hosts' response to the proteins encoded by retroviral genes determine the type of disease manifested [1]. Greater understanding of host immunity against retroviruses is pertinent in the era of a global HIV/AIDS epidemic.

The murine leukaemia viruses (MLVs) are members of the gamma-retrovirus genus of the Retroviridae family. The diseases caused by MLVs include lymphomas and leukaemias. Studies on Friend MLV led to the discovery of the archetypal regulation or restriction of viral infection by intrinsic host-cell defence mechanisms. Friend virus susceptibility factor (Fv1) is a dominant allele expressed in mice or cell lines adapted from specific species of mice that confers resistance to different MLV strains [2]. Hence, MLV-N strains (N-tropic MLVs) are unable to infect mice with the Fv^b/b ^genotype, and MLV-B strains are unable to infect mice with the Fv^n/n ^genotype. Mice with the Fv^n/b ^genotype are resistant to both strains of MLVs but are susceptible to viruses that are both N and B tropic, such as Moloney MLV (MLV-NB) [3]. Fv1 is a saturable gag-like element expressed from a murine endogenous retrovirus-L (MuERV-L) [4] closely related to the human ERV-L [5,6]. Fv1 blocks MLV virus prior to integration and does not block infection by other retroviruses [7]. On the virus side, historically, a single amino acid (aa) in the CA protein at position 110 is thought to determine if MLVs are B or N tropic [8,9]. However, more recent evidence suggests that residues up- or down- stream from this canonical site may also influence virus susceptibility to host immunity [10-12].

Less than a decade after Fv1 was cloned in 1996, the tripartite motif (TRIM) family of proteins were implicated in species-specific restriction of incoming retroviral CAs, initially referred to as the lentivirus restriction factor or Lv1 [13-15].

Unlike Fv1 restriction, the TRIM proteins have a broader reactivity and can act either before or after RT depending on the invading virus and host cell species. Rhesus TRIM5α (rhTRIM5α) can potently restrict HIV-1 infection; conversely human TRIM5α (huTRIM5α) has only minor effects on SIVmac and HIV-1 infectivity. HuTRIM5α, however, can restrict MLV-N virus replication (Ref1), but not MLV-B or-NB virus infection [14,16]. Interestingly, mutations in the B30.2/SPRY domain of huTRIM5α confer on it the ability to restrict MLV-B, SIVmac and HIV-2 viruses [10,17]. Yan and Kozak, [18] have described another CA mediated post entry resistance to the ecotropic MLV AKV in a murine cell line which is distinct from the classical Fv1 mediated restriction. This restriction is present in 3 out of the 4 major genera of *Mus *species, suggesting an extended role of Fv1 in *Mus *evolution and retroviral resistance pre-dating the classical Fv1 alleles determined for laboratory mice [19].

Evidence for Env mediated post entry restriction is gaining momentum. Previously, we described another lentivirus susceptibility factor termed Lv2; like Lv1 and Ref1, Lv2 is a post-entry restriction found in some human cells and their derivatives. However, in addition to CA, the virus Env also has a role in Lv2 restriction. The molecular clone non-restricted (MCN) virus derived from a tissue culture adapted HIV-2 isolate has a non-restricted phenotype, while the molecular clone restricted (MCR) virus derived from a primary isolate from the same patient is susceptible to Lv2. The Env mediated restriction of MCR is overcome by pseudotyping viral cores with VSV-G Env. Envelope and CA substitutions between MCR and MCN retroviral vectors pinpointed aa 74 in the Env and aa 207 in the CA as the viral determinants for Lv2 MCR restriction [20,21]. Another post-entry HIV-1 resistance factor, termed Lv3 [22], has been described in rhesus macaques and is dependent on CD4 and endogenous co-receptor viral delivery. Uchil *et al. *[23] have pseudotyped the avian leukaemia virus-A (ALV-A) Env onto HIV-1, MLV-N, and MLV-B cores and described novel effects on post viral entry in HEK293 cells transiently transfected with different murine and human TRIMs. Together, these studies suggest that the Env protein plays a role in mediating events post entry, at or during reverse transcription.

Given such subtleties in Env and CA determinants of intrinsic host cell resistance, we wished to determine if the Lv2 phenotype was operative for the MLVs in human cells. Because the huTRIM5α restriction of VSV-G Env pseudotyped MLV-N viruses is well characterised, we sought to determine the role, if any, of other human host restriction factors on the MLV-B and MLV-NB CAs. We tested our hypothesis by pseudotyping these CAs with two HIV-2 Envs that defined the Lv2 restriction in HeLa/CD4 cells.

This paper describes the restriction of MLV-B and MLV-NB viral cores in human cells when pseudotyped with HIV-2 [20,21] and the feline endogenous virus RD114 Envs. In comparison the same cores pseudotyped by the gamma-retroviral gibbon ape leukaemia virus (GALV), Amphotrophic (Ampho) MLV and the VSV-G Envs were relatively unrestricted in HeLa/CD4 cells.

Thus we provide evidence for a novel restriction of MLV-B and MLV-NB viruses in the human HeLa/CD4 cell line, which is dependent on the pseudotyping Env. This restriction is distinct from the previously characterised Ref1, Lv1, Lv2 and Lv3. Our data add another layer to the intricate puzzle of the relationships between different retroviruses and the myriad of host cell defences they encounter.

## Results

### HIV-2 Envs MCN and MCR mediate MLV-B and MLV-NB restriction in HeLa/CD4 cells which is rescued by different retroviral Envs

**Figure 1:**
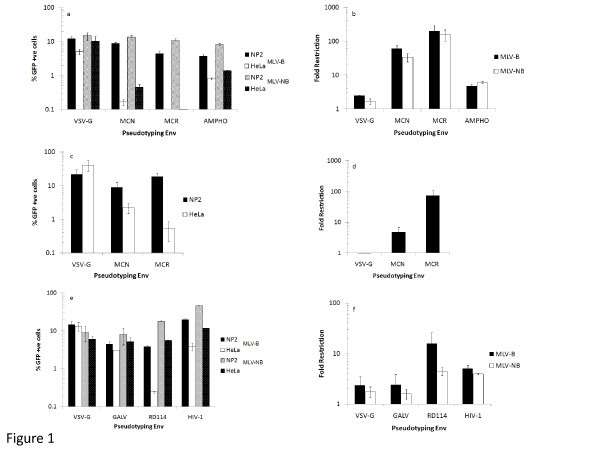
**HIV-2 Envs MCN and MCR mediate MLV-B and MLV-NB restriction in HeLa/CD4 cells, and the restriction is rescued by different retroviral Envs**. VSV-G, MCN, MCR and Ampho MLV Envs were pseudotyped onto MLV-B and MLV-NB cores, and viral titres were normalised on NP2/CD4/CXCR4 cells to 10% GFP +ve infection. (a) MCN and MCR pseudotyped MLV-B and MLV-NB viral vectors showed a restricted infection of HeLa/CD4 cells when compared to NP/CD4/CXCR4 cells, while Ampho pseudotyped vectors were relatively unrestricted. (b) Fold restriction is defined as the ratio of transduction of the non- restricted NP2/CD4/CXCR4 cells to the restricted HeLa/CD4 cell line for MLV-B and -NB viral cores. (c) MCN Env pseudotyped HIV-2 CA (RodA delta Env GFP) is not restricted on HeLa/CD4 cells while MCR is. (d) Fold restriction of HIV-2 CA RodA infectivity data in Figure 1c. (e) MLV-B pseudotyped with RD114 Env is restricted in HeLa/CD4 cells, GALV and HIV-1 Envs are not restricted. MLV-NB pseudotyped with RD114, GALV and HIV-1 Envs are not restricted in HeLa/CD4 cells. (f) Fold Restriction for the MLV-B and MLV-NB cored viral vectors in Figure 1e. Data represent the average of three or more independent experiments +/- S.E.M.

Previously we had demonstrated that an Env and a CA derived from an HIV-2 isolate, MCR, were determinants of Lv2 restriction in HeLa/CD4 cells. In contrast, the Env and CA derived from the MCN isolate are relatively unrestricted in the same cells. The susceptibility of MLV-N CA to Lv1 in human cells is well documented [14,16,24]. Here we sought to determine if MLV-B and MLV-NB cores would be restricted in an Lv2-like manner. However, unexpectedly, we observed that if MLV-B cores are pseudotyped with either the MCN or MCR HIV-2 Envs they both result in a restricted phenotype in HeLa/CD4 cells, but not in the NP2/CD4/CXCR4 (a permissive glioma cell line stably transduced with the HIV receptors [25]) cells (Figure 1a). Infection on NP2/CD4/CXCR4 is 60X (MCN) and 200X (MCR) greater than on the restricted HeLa/CD4 cells (Figure 1b). In contrast, the VSV-G and Ampho pseudotyped MLV-B vectors were relatively uninhibited in both cell lines, showing only 2X and 5X differences in infection (Figures 1a and 1b). Similar results were obtained for the MLV-NB CA pseudotyped with these Envs (Figure 1a). Infection of NP2/CD4/CXCR4 cells is 35X (MCN) and 160X (MCR) greater than on HeLa/CD4 cells. The VSV-G and Ampho pseudotyped MLV-NB vectors remain unrestricted, showing only 1X and 5X differences in infection of the two cell lines (Figure 1b). Thus we have shown that this restriction of MLV-B and -NB CAs is Env dependent. To further confirm that this restriction is also dependent on viral CA, we produced retroviral vectors with an unrelated HIV-2 CA, RodA. As expected, when we pseudotyped the RodA delta Env HIV-2 core with VSV-G, MCN and MCR Envs only the MCR Env was restricted in HeLa/CD4 cells (Figure 1c). Specifically, the VSV-G and the MCN pseudotyped vectors were unrestricted (0.6X and 4.7X difference between NP2 and HeLa cells), while the MCR Env showed 73X difference in infection (Figure 1d) similar to the Lv2 phenotype described previously [20]. Hence the restriction with MLV-B and MLV-NB is similar to Lv2 in its HeLa/CD4 specificity and is overcome by pseudotyping with VSV-G Env. Unlike Lv2, however, both the MCN and MCR Envs result in a restricted phenotype with these viral CAs. Importantly, these results show that HIV-2 Envs reveal a previously unreported restriction of MLV-B and MLV-NB cores in HeLa/CD4 cells.

Next we generated retroviral vectors with HIV-1 [26], GALV [27,28] and RD114 Envs [29]. These Envs were chosen to expand the receptor classes of these MLV CA for entry into human cells and have been well characterised as pseudotyping Envs for MLV cores [29,30]. GALV Env receptor, PiT1 [31] is closely related to the Ampho Env receptor PiT2 [32,33] and HIV-1 uses the same receptors as the HIV-2 Envs. Both MLV-B and MLV-NB pseudotyped viral cores were rescued from the restriction on HeLa/CD4 cells by the GALV and HIV-1 Envs (Figure 1e). The 2.4X and 5X difference in infection for GALV and HIV-1 are comparable to the VSV-G control (2.3X) (Figure 1f). Similarly, GALV (1.6X), HIV-1 (4X) and RD114 (4.5X) pseudotyped MLV-NB CAs were unrestricted (Figures 1e and 1f). Interestingly, the RD114 Env (which uses the neutral amino acid transporter abundantly expressed in human cells [29]) showed the highest levels of restriction compared to the VSV-G Env on the MLV-B CA (Figure 1e), with a 16X difference in infection of the two cell lines (Figure 1f). However, this level of restriction is less than that conferred by the HIV-2 Envs described above.

### Flow cytometry analysis demonstrates that NP2/CD4/CXCR4 and HeLa/CD4 cells express similar levels of receptors on their cell surface

**Figure 2:**
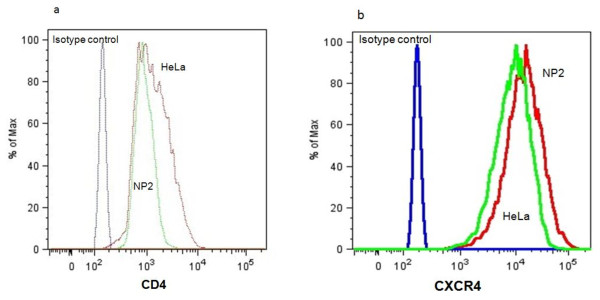
**Flow cytometry analysis shows equivalent numbers of CD4 and CXCR4 molecules on the surface of HeLa/CD4 and NP2/CD4/CXCR4 cells**. Cells were stained with Pacific blue conjugated mouse anti human CD4 and PE conjugated mouse anti human CXCR4, with appropriate isotype and unlabelled cell controls. (a) NP2/CD4/CXCR4 and HeLa/CD4 cells showed similar log shifts in CD4 fluorescent intensity, compared to isotype controls. (b) NP2/CD4/CXCR4 and HeLa/CD4 cells show similar log shifts in CXCR4 fluorescent intensity, compared to isotype controls.

The results above demonstrate that Env is a strong determinant of the ability of viral core to complete early events in replication. We next determined the HIV receptor levels of NP2 and HeLa cells to further support the notion that the restriction was not due to a difference in the expression of these receptors. We immunostained the cells with fluorescently labelled antibodies to CD4 and CXCR4 and used flow cytometry for their detection. These experiments show similar log shifts in fluorescent intensity for CD4 staining (Figure 2a) and CXCR4 staining (Figure 2b) on both HeLa/CD4 and NP2/CD4/CXCR4 cells. Hence, the restriction in HeLa/CD4 cells is not due to the reduced level of receptors on the surface of these cells in comparison to those seen on the unrestricted NP2/CD4/CXCR4 cells.

### QT-PCR data demonstrate that the MLV-B and MLV-NB restriction in HeLa/CD4 cells is post entry

We used QT-PCR to monitor retroviral transcription initiated after viral and plasma membrane fusion. HeLa/CD4 and NP2/CD4/CXCR4 cells were challenged with different pseudotyped viruses, at MOIs of 0.25 to 1, and incubated for 16 hrs or more to allow RT to proceed. Negative controls for infection included incubating virus for 5 minutes at 4°C or adding trypsin to target cells just prior to challenge (to strip the cell surface expression of receptors). In all cases viral inocula were DNase treated with 100 units DNase/ml of virus for 1 1/2 hrs 37°C and trypsinised off cells before lysis for DNA extraction. The extracted DNA was assessed by QT-PCR for late RT products. Primers and probes were designed to amplify the transfer plasmid pCNCG with an internal GAPDH control reaction. All data were normalized to the genomic GAPDH internal control (GAPDH amplification results are not shown).

**Figure 3:**
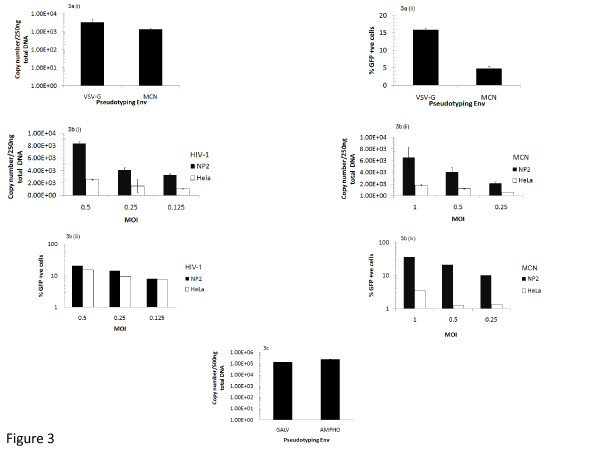
**QT-PCR analysis of late reverse transcripts generated in HeLa/CD4 and NP2/CD4/CXCR4 cells challenged with restricted and non-restricted MLV-B and MLV-NB viral vectors**. MLV-B and MLV-NB cored viral vectors pseudotyped with the different Envs described in Figure 1 were used to challenge confluent monolayers of HeLa/CD4 and NP2/CD4/CX/CR4 cells in 12 well trays with MOIs of 0.25-1. Controls for infection included incubation of infected cells for 5 minutes at 4°C or trypsinising target cells (to strip cell surface receptors off) just prior to infection. Normal infection involved incubating cell cultures O/N (12-16 hrs) to 48 hrs at 37°C (for the FACS equivalent). Total extracted DNA was normalised to 250 or 500 ng/PCR reaction. Primers and probes were designed to amplify the eGFP target, with an internal genomic GAPDH control. The data shown have been normalised to the GAPDH amplification and for the background level of transcripts seen in uninfected cell controls for each experiment. (a) (i) MLV-B viral vectors pseudotyped with VSV-G Env, MLV-NB viral vectors pseudotyped with HIV-2 MCN Env, cells were incubated 5 minutes at 4°C and overnight 37°C incubation, data shown are for RT transcripts detected after overnight 37°C incubation minus the number detected for the 5 minutes at 4°C incubation for each Env, MOI of 1 (ii) Corresponding infection data (cells incubated for 48 hrs) for the QT-PCR shown in (i); (b) (i and ii) HIV-1 and HIV-2 MCN pseudotyped MLV-NB CA, titrated at MOIs 0.25-1 (iii and iv) Corresponding infection data (cells incubated for 48 hrs) for the QT-PCR shown in (i and ii); (c) MLV-NB viral vectors pseudotyped with GALV and Ampho Envs, cells were incubated O/N, MOI of 0.3. Data shown for the QT-PCRs are representative of 3 or more independent experiments. Error bars represent +/- STDEV within 1 experiment.

Figure 3a (i) shows that the level of newly transcribed DNA transcripts after O/N incubation 37°C on HeLa/CD4 cells were equivalent (MOI 1). The corresponding infectivity data (Figure 3a (ii)) show that the MCN pseudotyped virus is, however, much less infectious compared to VSV-G Env pseudotyped virus. Figure 3b (i and ii) shows that regardless of viral Env, pseudotype infection of NP2 cells always resulted in greater numbers of RT transcripts compared to infection of HeLa cells. Importantly, however, a comparison of transcripts produced after challenge of HeLa cells resulted in the same number of transcripts regardless of whether the CA was pseudotyped with restrictive or non-restrictive Envs. The levels of RT transcripts detected correspond to the different MOIs used to challenge cells. The corresponding infectivity data (Figure 3b (iii and iv)) confirm that in NP2 cells the transcripts result in permissive titratable infection for both Envs, but in HeLa cells, while the HIV-1 Env virus results in efficient infection, the HIV-2 MCN virus is restricted.

Similar results were observed with the different pseudotyped combinations described in this paper. In Figure 3c we show that the GALV and Ampho Envs produce similar levels of transcripts in HeLa cells.

In summary, the data showed that viral transcripts were overall reduced in HeLa cells when compared to NP2 cells, in a dose dependent manner. Importantly, these QT-PCR data show that the number of RT transcripts for the restricted Envs are similar to the non-restricted Envs in HeLa cells. These data thus confirm that the restriction seen in HeLa cells is not due to a block of viral entry at the plasma membrane, but is at a post entry post RT level.

### The observed restriction to MLV-B and MLV-NB in HeLa/CD4 cells is not mediated by huTRIM5α

**Figure 4:**
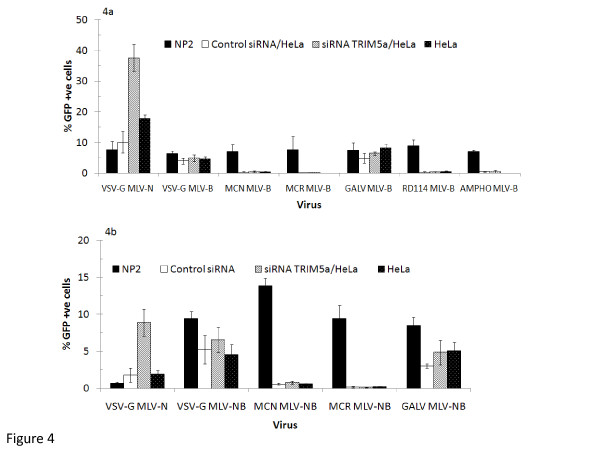
**siRNA knockdown of huTRIM5α reduces VSV-G MLV-N restriction in HeLa/CD4 cells but has no effect on MLV-B and MLV-NB**. (a) VSV-G pseudotyped MLV-N viral vectors were used to challenge HeLa/CD4 cells that were pre-treated with 50 pmol siRNA huTRIM5α, control siRNA and untreated cells. As expected, treatment with siRNA huTRIM5α resulted in a relief of the restriction of MLV-N cores, while treatment of cells with the control siRNA had no effect on the transduction. MLV-B viral vectors pseudotyped with restricted (MCN, MCR, RD114) and non restricted (VSV-G, Ampho and GALV) Envs were used to infect HeLa/CD4 cells that were pre-treated with the optimal siRNA huTRIM5α concentration determined for MLV-N (50 pmol), control siRNA or non-siRNA treated. Untreated NP2/CD4/CXCR4 cells were also infected as a control. The siRNA huTRIM5α treated cells showed no relief of restriction of the MLV-B viral vectors. (b) As for Figure 4a, but with MLV-NB virus. Data represent the average of three or more independent experiments +/- S.E.M.

HuTRIM5α has been well documented as a restriction to MLV-N, but not MLV-B and MLV-NB pseudotyped with a VSV-G Env. We down regulated TRIM5α production using specific siRNA knockdown to determine whether or not it had a role in the restricted phenotype described here. As shown by others, siRNA knockdown of huTRIM5α relieved the restriction in HeLa/CD4 (with an increase from 18% to 38% in transduction) of VSV-G pseudotyped MLV-N viral vectors, but not with control siRNA (Figure 4a). Similar results were obtained on NP2/CD4/CXCR4 cells (7.6% to 26.4% (data not shown). By comparison, siRNA knockdown of huTRIM5α did not affect the HIV-2 (MCN, MCR) and RD114 Env mediated restriction of MLV-B CAs in the HeLa/CD4 cells (Figure 4a). The results were similar for MLV-NB cored viruses (Figure 4b). Therefore, the restriction of MLV-B and MLV-NB cores, pseudotyped by HIV-2 and RD114 Envs, is not due to the direct activity of huTRIM5α on incoming CAs.

## Discussion

Here we describe a novel post entry restriction in HeLa/CD4 cells of MLV-B and MLV-NB viral cores when pseudotyped with two HIV-2 Envs and one RD114 Env, demonstrating that human cells have factors other than TRIM5α which inhibit MLV infection.

Studies of the post-entry actions of host cellular factors to block specific retroviral CAs from establishing active infections have provided a greater understanding of different retroviral/host interactions and their co-evolution. These findings imply that retroviruses evolve envelopes to avoid innate host defences that target retroviral infections.

While the study of innate retroviral host cell immunity to incoming CAs has typically employed VSV-G Env pseudotyped vectors, there is a growing body of evidence to suggest that the viral entry route also contributes to the outcome of infection [17,20-22]. Previously, we demonstrated the Lv2 restriction in HeLa/CD4 cells to a molecular clone of an HIV-2 isolate, MCR. Unlike Lv1 and Fv1, Lv2 is determined by Env in addition to CA. Lv2 restriction is overcome by the substitution of MCR Env/CA with either their non-restricted MCN equivalents or with VSV-G Env [20,21]. Similarly the *Minr *resistance factor [18] has recently been shown in cells from the African pygmy mouse *M. minutoides *against non lab-adapted strains of AKV ecotropic MLVs. *Minr *also acts post viral entry, and is distinct from any of the Fv1 allelic restrictions described previously. It was noted that lab-adapted MoMLV and FRMLV57 Envs displayed a 10- fold greater titre than AKV Envs in NIH3T3 cells when compared to *M. minutoides *cells, and speculated that like Lv2 there may be an Env dependent pre-RT determinant of *Minr *resistance [18].

Given the extent to which MLV-B, MLV-NB and MLV-N CAs have been investigated and their differences in susceptibility to Fv1 and huTRIM5α restriction [9-12,16,17,23], we specifically determined whether or not the delivery of MLV-B and -NB CAs into human cells by the MCN and MCR HIV-2 Envs would affect transduction in a manner consistent with the Lv2 restriction.

Surprisingly, our data revealed a restriction distinct from Lv-2 because both MLV-B and MLV-NB cored viruses were restricted in HeLa/CD4 cells if pseudotyped by either HIV-2 Env. GALV and VSV-G Envs rescued this restriction. By comparison, the RD114 Env pseudotyped MLV-B virus was restricted, but not to the levels seen with the HIV-2 Envs. It is interesting that the MLV-NB core was not restricted if delivered by an RD114 Env. This difference may reflect the adaptive changes MLV-NB has undergone in relation to both MLV-N and MLV-B typified by its lack of sensitivity to both Fv1 and huTRIM5α activity [9-12,16,17,23].

Ampho Env pseudotyped MLV-B and MLV-NB cores were relatively more restricted than GALV and less restricted than RD114 Envs (Figures 1a, b, e and 1f). It is well documented that GALV Env pseudotyped retroviral vectors show higher transduction efficiencies than Ampho Env vectors on human cells [34]. The prevailing hypothesis is that the discrepancy in target cell receptor density levels of PiT1 [31] (GALV receptor) and PiT2 (Ampho receptor) contribute to these differences [33,35]. However, we show by QT-PCR that there is no difference in the levels of late RT transcripts between GALV and Ampho Env vectors in HeLa/CD4 cells (Figure 3c). Hence the difference in susceptibility is unlikely to be entirely due to just receptor density levels and perhaps reflects subtleties in the adaptation to different host species.

Both HIV-1 and HIV-2 use CD4 and CXCR4 as receptors for entry. Unlike the HIV-2 Envs, the HIV-1 Env pseudotyping did not restrict MLV CAs in HeLa/CD4 cells. Fluorescent receptor labelling with detection by flow cytometry revealed similar amounts of both molecules on the surface of both HeLa/CD4 and NP2/CD4/CXCR4 cells (Figures 2a and 2b). These data suggest that the restriction we describe is not due to a lack of entry because of differences in receptor density. Furthermore, the QT-PCR data demonstrates that the levels of RT transcripts for the restricted HIV-2 MCN and non-restricted HIV-1 pseudotyped viruses were similar (Figures 3b (i and ii)).This conclusion is further supported by the QT-PCR data which showed that the levels of transcripts in the HeLa/CD4 cells were similar for the different restricted and non restricted Envs (Figures 3a-c). These data combined suggest a post entry, post RT mechanism of action involved in the HeLa/CD4 cells.

Given the data presented here and the increasing evidence that host cell restriction is not solely dependent on the susceptibility of an incoming viral CA; a central question remains one of why/how? HIV-1 has rapidly adapted from its SIV progenitor and led to a world-wide HIV epidemic, whereas the closely related HIV-2 has not. Previous studies have shown that HIV-2 replication in primary macrophages is characterised by what is believed to be a 'latent state' when compared to continuous HIV-1 production [36]. It is possible that the adaptations of HIV-1 Env are more successful than those of HIV-2, with HIV-1 adapting its route of entry to over-ride some of the innate human defences it has hitherto encountered.

Since MLV-N is restricted by huTRIM5α, we used siRNA knockdown experiments to determine if huTRIM5α had a role in this novel restriction of MLV-B and MLV-NB CAs. Our data clearly demonstrated that while the siRNA huTRIM5α treatment relieved restriction of VSV-G pseudotyped MLV-N virus, this was not the case with MLV-B and MLV-NB cores (Figures 4a and 4b).

In summary, our data indicate that there is a novel post entry restriction to MLV-B and -NB CAs in human cells and provide more evidence on Env contribution of CA restriction by innate retroviral responses.

## Methods

### Cell lines

HeLa/CD4 (human squamous epithelial carcinoma) cells and human glioma cell NP2/CD4/CXCR4 [25] were maintained in Dulbecco's modified essential medium (DMEM) supplemented with 10% foetal calf serum (FCS), 60 μg of penicillin/ml, 100 μg of streptomycin/ml, and 1 mg of G418/ml. NP2/CD4/CXCR4 DMEM also contained 1 μg of puromycin/ml. *Mus dunni *tail fibroblasts (MDTF) and 293 T cells were maintained in DMEM supplemented with 10% FCS, with the penicillin and streptomycin concentrations as described above.

### Expression Plasmids

The mammalian expression plasmids used to generate retroviral vectors in this study included: pMDG VSV-G Env; pMP11-MCRenv, pMP11-sMCNenv [20], GALV Env [37], Ampho Env [38], RD114 Env, pCIG3-N MLV-N core, pCIG3-B MLV-B core, pCIG3 MLV-NB core, pCNCG transfer plasmid encoding enhanced GFP (eGFP) [39].

### Production of retroviral vectors

Retroviral vectors were produced using a three plasmid transfection system in 293 T cells as described previously [39]. 293 T cells were passaged 24 hrs prior to transfection and seeded at 2.5-3 × 10^6 ^cells in a 10-cm^2 ^tissue culture dish. The polyethylenimine (PEI, Sigma) transfection reagent was used in all transfections (stock 1 mg/ml). Plasmids were transfected at a ratio of 1:2:3 Env:Core:Transfer. A total of 18 μg DNA was combined with 60 μl of 1 mg/ml PEI in 900 μl of serum-free DMEM (Gibco, Invitrogen Corp.). The suspension was vortexed briefly, incubated for 10 mins at room temperature and added drop-wise to the 293 T cells. Transfected cells were incubated for a minimum of 5 hrs or O/N at 37°C (5% [vol/vol] CO_2_) in a humidified incubator; transfection supernatant was removed and cells were overlaid with 10 ml of fresh 10% FCS DMEM for 48 and 72 hrs harvests of viral vector supernatants.

Transfection supernatants were filtered through a 0.45 μm-pore-size filter (Millipore, Bedford MA, USA) and viral vector-containing medium stored in 500 μl aliquots at -80°C until required.

### Infection assays

Infection assays of different cell lines were carried out by seeding cells at a density of 2 × 10^4 ^cells/well in 48-well cell culture plates in a total volume of 500 μl growth DMEM, one day prior to challenge with the different pseudotyped retroviral vectors. The next day viral vectors were applied to the cells, in a total volume of 200 μl/well (supplemented with 5% FCS DMEM). The medium was removed 2-3 hrs later and replaced with fresh DMEM and the cells were cultured for a further 48 hrs before analysis for the expression of GFP positive cells by flow cytometry with a FACScan (Becton Dickinson). Cells were fixed in 3 ml 3.7% formaldehyde/PBS for 3 mins at RT, pelleted by centrifugation at 1500 RPM, washed once in 1×PBS and resuspended in 200 μl PBS for flow cytometry. MLV-B and MLV-NB cored virus stocks were titrated and normalised on NP2/CD4/CXCR4 cells and MLV-N virus stocks on MDTF cells before their addition to the target cell lines. Fold restriction (x-fold) is calculated as the proportion of GFP +ve cells in a non-restricted infection/restricted infection.

### siRNA knockdown of huTRIM5α

#### Optimisation of HiperFect: siRNA concentrations

HeLa/CD4 cells were seeded in a 6-well plate at a density of 2 × 10^5 ^cells/well in a total volume of 2 ml, one day prior to transfection. HuTRIM5α and a scrambled control siRNA were titrated with varying volumes of HiPerFect transfection reagent as per manufacturer's guidelines (QIAGEN, UK). In short, HiPerfect and siRNAs were individually diluted in serum free OPTIMEM, then mixed and incubated for 10 min RT. siRNA transfection mixtures were added drop wise to HeLa/CD4 cells and incubated in a total of 1 ml serum free OPTIMEM for 30 mins at 37°C in a humidified incubator. Cells were overlaid with 1.5 ml fresh 10% FCS DMEM and incubated O/N. Non siRNA treated HeLa/CD4 cells were included as controls. The next day, siRNA transfection supernatants were removed and cells were challenged with VSV-G pseudotyped MLV-N viral vectors. Infected cells were incubated for a further 48 hrs before flow cytometry analysis for the expression of GFP.

HeLa/CD4 cells were seeded in a 48-well plate at a density of 2 × 10^4 ^cells/well in a total of 500 μl, one day prior to transfection. Optimised HiPerFect and siRNA huTRIM5α and a control siRNA were transfected as above and the next day transfected cells were infected with equal amounts of MLV-B or MLV-NB viral vectors pseudotyped with the different Env (VSV-G Env MLV-N control) which were previously normalized on NP2/CD4/CXCR4 cells. NP2/CD4/CXCR4 cells were infected with equal amounts of virus as a positive control for viral infectivity. Infected cells were incubated for a further 48 hrs before flow cytometry analysis for the expression of GFP.

#### Detection of CD4 and CXCR4 expression on HeLa/CD4 and NP2/CD4/CXCR4 cells

HeLa/CD4 and NP2/CD4/CXCR4 cells were seeded in 6 well plates at a density of 5 × 10^5 ^cells/well in a total of 2 ml DMEM culture medium. The following day, cells were removed from the plate with 5 mM EDTA, pelleted and incubated with 5 μl/100 μl PBS Pacific blue conjugated mouse anti human CD4 and PE conjugated mouse anti human CXCR4. Mouse anti human IgG isotype controls were used for each antibody and unlabelled cells were also included as controls. Cells were stained at RT for 1 hr then washed and fixed for flow cytometry analysis.

### Quantitative late PCR

HeLa/CD4 and NP2/CD4/CXCR4 cells were seeded in a 12-well plate at a density of 8 × 10^4 ^cells/well in a total volume of 1 ml, one day prior to infection. The following day, the spent medium was replaced with up to 1 ml of fresh medium containing 4 × 10^4 ^-1.6 × 10^5 ^IUs/ml (MOI of 0.25-1) of viral vectors. All vector stocks were pre-treated with 100 U of DNaseI/ml virus for 1 hr 30 mins at 37°C ([40]) to degrade excess plasmid DNA from the transfection stage. Normal infections were incubated O/N 37°C. Controls for infection included trypsinising the cells just prior to infection in 10× Trypsin/EDTA for 15 minutes at 37°C (to strip off the cell surface receptor expression) or absorbing virus at 4°C for 5 minutes with immediate lysis. All virus incocula were trypsinised off infected cells after the appropriate incubation times and temperatures, followed by 2× washes in 10× Trypsin/EDTA, before lysis and DNA extraction with a QIAamp blood DNA mini kit according to the manufacturer's protocol (QIAGEN, UK).

The isolated DNA was subjected to quantitative PCR (QT-PCR) to determine the late GFP RT transcripts present. Each 25 μl reaction mixture for the detection of late GFP RT DNA transcripts contained the following components: 1× MegaMix PCR buffer (Microzone Limited), 400 nM forward primer (5'-CAACAGCCACAACGTCTATATCAT-3'), 400 nM reverse primer (5'-ATGTTGTGGCGGATCTTGAAG-3'), 100 nM probe (5'-6-carboxyfluorescein- CCGACAAGCAGAAGAACGGCATCAA -6- carboxy-tetrafluorescein-3'), and 500 ng of total DNA. As a control for the total amount of DNA used in each reaction, GAPDH forward and reverse primers and a CY5 probe were also included in each sample tested. A standard curve was prepared with the pCNCG transfer plasmid in a background of 200 ng of salmon sperm carrier genomic DNA (data not shown).

PCR amplifications, data acquisition, and analysis were performed with the ABI PRISM 7500 sequence detection system. PCR conditions consisted of one cycle of denaturation (95°C for 5 min) followed by 40 cycles of amplification (95°C for 15 s, 60°C for 1 min).

## Abbreviations

MLV: murine leukaemia virus; HIV-1 and 2: human immunodeficiency virus types 1 and 2; VSV-G: vesicular stomatitis virus G protein; Fv1: Friend virus susceptibility gene 1; Ref1: retrovirus restriction factor 1; Lv1-3: lentivirus restriction factors 1-3; TRIM: tripartite motif; Env: envelope; CA: capsid; RT: reverse transcription; QT-PCR: quantitative PCR; siRNA: small interfering RNA; MCR: molecular clone restricted; MCN: molecular clone non restricted; RD114: feline endogenous retrovirus; GALV: gibbon ape leukaemia virus; Ampho: Amphotrophic; aa: amino acid; MDTF: *Mus dunni *tail fibroblasts; GFP: green fluorescent protein; MOI: multiplicity of infection.

## Competing interests

The authors declare that they have no competing interests.

## Authors' contributions

NMMO designed and carried out experiments and drafted the manuscript.

RT carried out experiments and drafted the manuscript. AM conceived the study and participated in experimental design and co-ordination and helped draft the manuscript. All authors read and approved the final manuscript prior to submission.
